# A Good Suggestion

**Published:** 1883-06

**Authors:** 


### A GOOD SUGGESTION.

Prof. Taft, in the June number of the *Dental Register*, says—

"The journalistic interests of the dental profession of the country have assumed such an importance and magnitude as to warrant associate and conjoined consideration on the part of those now immediately connected with and conducting its affairs."

He suggests that the editors of the various dental journals of the country hold a meeting during the sessions of the American Dental Association at Niagara, and that a permanent organization be formed. Every profession must be greatly dependent upon its literature for its healthy tone, and the improvement and growth of the various journals devoted to dentistry might be materially assisted in many ways by a thorough understanding between and fraternal co-operation among their conductors. Especially should this be beneficial to the younger journalists, and we therefore heartily second the proposition of the *Register*, and suggest that Professor Taft, as the undisputed Dean of the corps of dental editors, issue a formal call for such meeting.

